# Laminin α4 Deficient Mice Exhibit Decreased Capacity for Adipose Tissue Expansion and Weight Gain

**DOI:** 10.1371/journal.pone.0109854

**Published:** 2014-10-13

**Authors:** Marcella K. Vaicik, Jill Thyboll Kortesmaa, Sofia Movérare-Skrtic, Jarkko Kortesmaa, Raija Soininen, Göran Bergström, Claes Ohlsson, Li Yen Chong, Björn Rozell, Margo Emont, Ronald N. Cohen, Eric M. Brey, Karl Tryggvason

**Affiliations:** 1 Department of Biomedical Engineering, Illinois Institute of Technology, Chicago, Illinois, United States of America; 2 Division of Matrix Biology, Department of Medical Biochemistry and Biophysics, Karolinska Institutet, Stockholm, Sweden; 3 Center for Bone and Arthritis Research, Institute of Medicine, The Sahlgrenska Academy, Gothenburg University, Göteborg, Sweden; 4 Department of Molecular and Clinical Medicine, Sahlgrenska University Hospital, Göteborg, Sweden; 5 Cardiovascular and Metabolic Disorders Program, Duke-NUS Graduate Medical School, Singapore, Singapore; 6 Clinical Research Center and Division of Pathology, Infectious Diseases, Department of Immunology, Microbiology, Pathology, and Infectious Diseases, Karolinska Institutet, Huddinge University Hospital, Stockholm, Sweden; 7 Section of Endocrinology, Department of Medicine, University of Chicago, Chicago, Illinois, United States of America; 8 Research Service, Hines Veteran Affairs Hospital, Hines, Illinois, United States of America; University of Minnesota, United States of America

## Abstract

Obesity is a global epidemic that contributes to the increasing medical burdens related to type 2 diabetes, cardiovascular disease and cancer. A better understanding of the mechanisms regulating adipose tissue expansion could lead to therapeutics that eliminate or reduce obesity-associated morbidity and mortality. The extracellular matrix (ECM) has been shown to regulate the development and function of numerous tissues and organs. However, there is little understanding of its function in adipose tissue. In this manuscript we describe the role of laminin α4, a specialized ECM protein surrounding adipocytes, on weight gain and adipose tissue function. Adipose tissue accumulation, lipogenesis, and structure were examined in mice with a null mutation of the laminin α4 gene (*Lama4^−/^* ^−^) and compared to wild-type (*Lama4^+/+^*) control animals. *Lama4^−/^* ^−^ mice exhibited reduced weight gain in response to both age and high fat diet. Interestingly, the mice had decreased adipose tissue mass and altered lipogenesis in a depot-specific manner. In particular, epididymal adipose tissue mass was specifically decreased in knock-out mice, and there was also a defect in lipogenesis in this depot as well. In contrast, no such differences were observed in subcutaneous adipose tissue at 14 weeks. The results suggest that laminin α4 influences adipose tissue structure and function in a depot-specific manner. Alterations in laminin composition offers insight into the roll the ECM potentially plays in modulating cellular behavior in adipose tissue expansion.

## Introduction

Obesity continues to increase globally in both industrialized and developing countries, with the World Health Organization reporting that worldwide obesity rates have doubled since 1980. Over 1 billion adults 20 years of age or older are overweight with an estimated 500 million of these defined as obese. Obesity is a major risk factor for several chronic diseases, contributing to a dramatic increase in morbidity and mortality due to type 2 diabetes, hypertension, heart disease, and other malignancies [1]. These issues with weight control result in a greater than $190 billion annual burden on US healthcare, and it has been suggested that the negative effects of obesity outweighs the positive effects of smoking cessation on the overall health of the population [2].

The ability to modulate adipose tissue expansion and function could be a tremendous benefit to healthcare. Adipocytes are complex endocrine cells involved in insulin sensitivity, feeding behavior, and neuroendocrine function. A number of soluble hormones and growth factors have been shown to have an influence on adipose tissue insulin sensitivity and expansion. However, little attention has been paid to the contribution of interactions between adipocytes and the extracellular matrix (ECM) to this process. Adipocytes are in constant contact with a network of insoluble proteins and polysaccharides known as the ECM. ECM interactions in adipose tissue can be divided into two major categories: (1) interactions with basement membranes (BM), a thin layer sheet surrounding differentiated adipocytes, and (2) interactions with stromal, or interstitial ECM that occur when adipocytes invade the tissue stroma during adipose tissue expansion. In other tissues, it is well-known that interactions with the ECM can regulate cell growth, differentiation, and migration, and influence tissue development and repair [3]–[7]. However, little is known about the influence of the ECM on adipose tissue.

While adipose tissue-ECM interactions have not been studied extensively, there is some evidence that the ECM plays an important role in regulating adipogenesis and adipocyte function [8]. The synthesis and remodeling of ECM molecules is enhanced during adipogenesis [9,10]. Differentiating preadipocytes degrade the local matrix, invade the surrounding stroma, and then synthesize new ECM components as they mature [11]. The ECM surrounding preadipocytes transitions from fibronectin-rich to laminin-rich during differentiations primarily through an increase in an α4 chain containing BM protein laminin [12]. In cell culture models, a preadipocyte cell line has been shown to express laminin, entactin and collagen IV during differentiation [13]. Laminins LN-411 and LN-421, are laminin isoforms consisting of a triple helix of the α4, β1 or β2 and γ1 chains, are expressed in excess to other isoforms at this time [14]. The α4 chain of laminin is present in the BM surrounding fully differentiated adipocytes and is upregulated during differentiation. However, the potential importance of the α4 chain laminins in the BM surrounding adipocytes has not been elucidated.

In this study, mice with a null mutation of the laminin α4 gene (*Lama4^−/^* ^−^) were used to examine a potential role for α4 chain laminins in adipose tissue expansion and function. Weight gain, adipose tissue function and adipose tissue structure were examined in *Lama4^−/−^* mice and compared to wild-type control animals. The *Lama4^−/−^* mice were found to be resistant to age-related and diet-induced obesity, and exhibited a depot-specific change in adipose tissue structure, volume and function.

## Material and Methods

### Animals, diets and housing

The generation of laminin α4 null mice (*Lama4^−/−^*) was previously described [15]. The mice were backcrossed to C57 BL/6 mice (Charles River) for more than 10 generations. Mice were fed a standard diet, or a high-fat diet containing 45 energy % fat (D12451, Research Diets), beginning at 4 weeks of age. The animals were fed *ad libitum* and their food was weighed weekly. The mice were given a food refill up to 500 g after each weighing. The amount of food consumed was divided by the number of animals in a cage as an estimate of intake.

All animal procedures were approved by the IACUC at Karolinska Institutet or the University of Chicago. The animals were housed either in mixed cages (two *Lama4^−/−^* and two wild-type control animals) or in cages with only *Lama4^−/−^* mice or wild-types, in order to rule out the possibility that the weight differences observed were due to differences in dominance behavior. No differences were observed due to housing conditions.

### Immunostaining

For immunostaining in mouse tissues, animals at 16 weeks of age were sacrificed and tissue harvested. Samples were placed in TissueTek (Sakura) in plastic molds and frozen in isopentane cooled to its freezing point. Cryosections of 8–12 µm in thickness were made at −38°C. The sections were allowed to dry for 1 hour at room temperature and then fixed in acetone for 10 minutes before staining, except for antibody to laminin α4, where the sections were additionally treated for 5 minutes in boiling 1 M Urea and washed in distilled water.

The antibodies used anti-nidogen/entactin (MAB 1946, Chemicon), anti-collagen type IV (polyclonal # AB756P, Chemicon), anti-perlecan (clone HK-102, Seikagaku Corp), anti-laminin α1 (clone 198 (35)), anti-laminin α2 (clone 4H8-2), anti-laminin α4 (polyclonal S8 (36)), anti-laminin α5 (serum 405). Secondary antibodies were FITC- or Cy3- conjugated and purchased from Jackson ImmunoResearch Laboratories, Inc. Tissue sections were examined with a Leica MDRB microscope (Leica) and pictures were taken with a Hamamatsu digital camera with Openlab (Improvision) software. Digital images were further processed with Photoshop 5.0 (Adobe).

### Liver histopathology

Livers were harvested from 40 week old Lama4^−/−^ mice for histopathological evaluation (10 *Lama4^−/−^* mice on both diets). For histological staining the tissue samples were fixed in 10% neutral buffered formalin, paraffin-embedded and stained according to standard protocols. Tissue sections were examined with a Leica MDRB microscope (Leica) and pictures were taken with a Hamamatsu digital camera with Openlab (Improvision) software. Digital images were further processed with Photoshop 5.0 (Adobe).

### Adipose tissue depot structure

*Lama4^+/+^* and *Lama4^−/−^* mice were fed a standard diet. At 14 weeks of age mice were sacrificed. Epididymal and subcutaneous fat depots were harvested, and the mass assessed. Mass of adipose tissues from each depot was normalized to the total individual animal weight the depot was harvested from using equation (1). The normalized % fat pad weight takes into account variation introduced from individual total animal weights. 
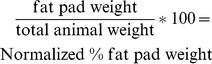
(equation 1)


A portion of each fat pad type was then placed in formaldehyde and paraffin embedded. Samples were sectioned and stained with hematoxylin and eosin. Five images were taken with an Axiovert 200 inverted microscope using a 5× objective (1.3 µm/pixel) (Carl Zeiss MicroImaging, Inc., Thornwood, NY) for each fat pad. The images were used to manually measure the diameters of individual adipocytes using AxioVision (Carl Zeiss MicroImaging).

**Lipogenesis.** *Lama4^+/+^* and *Lama4^−/−^* mice were fed a standard diet. At 14 weeks of age mice were sacrificed. Fat pads were harvested and weighed prior to functional analysis with a lipogenesis assay. The assay was performed as described previously [16]. Briefly, adipocytes were isolated from the harvested fat pads by collagenase digestion and centrifugation. Isolated adipocytes were incubated with radioactive glucose in Krebs-Ringer bicarbonate containing 10 nm insulin and 1% (w/v) BSA. The lipid fraction was extracted and radioactivity in the triglyceride fraction measured.

### Statistics

The repeated measures of animal weights over time and food consumption over time were analyzed with ANOVA followed with Tukey test. Student's *t* test was used for all other statistical analysis (two-tailed), p<0.05 was considered significant.

## Results

### Adipose tissue composition of *Lama4* ^−/−^ mice

**Figure 1:**
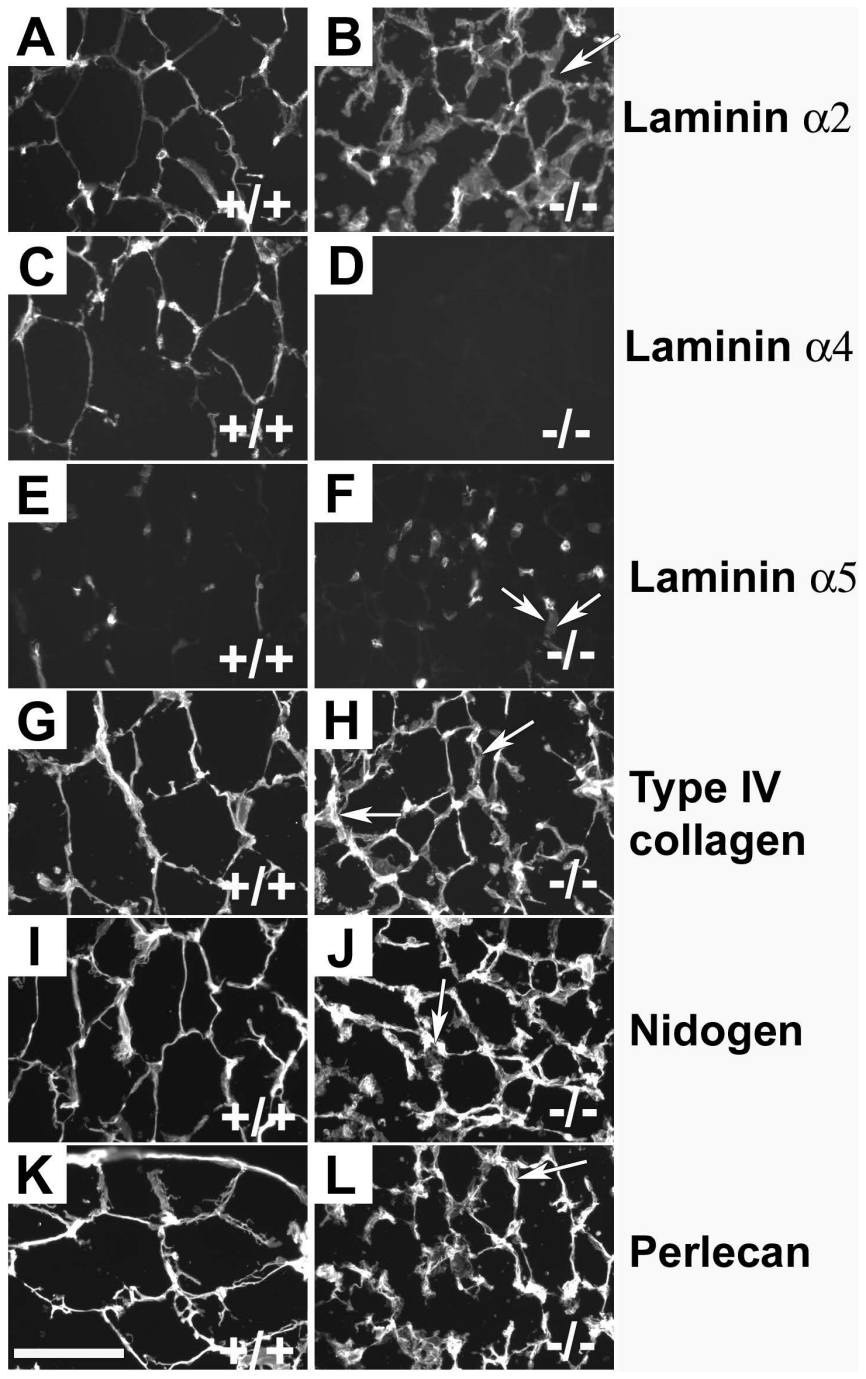
Immunostaining of WAT. The capillaries and BMs of adipocytes of wild-type control and *Lama4Lama4^−/−^* mice were positive with antibodies against laminin α2 (A, B). Laminin α4 antibodies stained both adipocytic BMs and capillaries in wild-type controls (C), while no staining was seen in mutants (D). The laminin α5 antibody (E, F) stained capillaries in *Lama4^−/−^* and wild-type controls, but not the adipocytic BM. Antibodies against type IV collagen (G; H), nidogen (I, J) and perlecan (K, L) all stained both capillaries and adipocytic BMs. Laminin α1 staining was completely negative (not shown). Arrows indicate a few locations where the adipocytic BM of the *Lama4^−/−^* mice appeared slightly thickened. Bar 100 µm.

Immunofluorescence staining was first performed to compare BM composition surrounding adipocytes in *Lama4^−/−^* and *Lama4^+/+^* mice. Staining was performed for known adipose tissue BM proteins, including the α1,α2, α4 andα5 chains of laminin, type IV collagen, nidogen and perlecan. In wild-type control mice the α2 and α4 chains of laminin were present in the BM surrounding mature adipocytes (Figure 1). Laminin α5 was not observed in mouse adipocyte BM. Type IV collagen, perlecan and nidogen were present the murine pericellular adipocyte BM. When examining *Lama4^−/−^* BM, the only difference in composition was the complete absence of laminin α4. All other BM proteins present in the adipose tissue BM of wild-type control mice were also observed in the pericellular BM of *Lama4^−/−^* mice. The adipocyte BM appeared somewhat thicker in the *Lama4^−/−^* mice.

**Figure 2:**
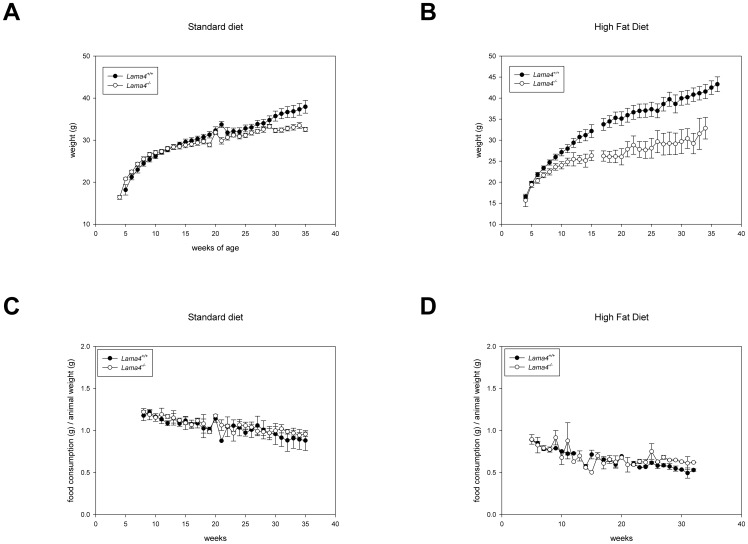
The weight difference of 16 male *Lama4^−/−^* mice and 12 wild-type controls were statistically significantly (p = 0.002). (A). When fed a high-fat diet (B), 8 male wild-type control mice gained statistically significant (p<0.001) more weight than the 8 *Lama4^−/−^* mice. The weekly intake of standard food (C) and high fat diet (D) related to the weight of the animals were not significantly different.

**Lama4^−/−^ mice are resistant to age and diet induced obesity.** The birth weight of laminin α4 deficient mice was ∼10% lower than wild-type control littermates as previously reported [15]. At the time of weaning (3–4 weeks), no difference in weight was observed between *Lama4^−/−^* or wild-type control animals (*Lama4^−/−^* 20.8±0.26 g, n = 16; *Lama^+/+^* 18.2±1.05 g, n = 11). Animal weights were monitored first on a standard diet. A statistical difference in weight was observed between wild-type control and *Lama4^−/−^* animals (Figure 2A; p = 0.002). *Lama4^−/−^* weighed less than wild-type control animals from 16 weeks on and the difference between knockout and wild-type control animal weights increased steadily over time.

To further investigate weight gain in these animals, mice were supplied a high-fat diet starting at four weeks of age. *Lama4^−/−^* animals gained weight at a much lower rate and differences were observed from wild-type control animal weights within 4 weeks on this diet (*Lama4^−/−^ 22.45*±0.73 g, n = 8; *Lama^+/+^ 24.69*±0.61 g, n = 8; Figure 2B). This difference continued to increase over time with *Lama4^−/−^* exhibiting slow weight gain even on the high-fat diet. In fact, at 34 weeks of age, there was no difference in the average weight of *Lama4^−/−^* mice whether they were on standard or high-fat diets (standard diet 32.6±0.5 n = 12 vs high-fat diet 32.8±2.57 n = 3). Wild-typetype control animals rapidly gained weight on the high fat diet resulting in dramatic differences from *Lama4^−/−^* at 13 weeks (*Lama4^−/−^* 25.4±0.89 g, n = 8; *Lama^+/+^* 30.7±1.27 g, n = 8;). A statistical difference in weight was observed between wild-type control and *Lama4^−/−^* animals on high fat diet (Figure 2B; p<0.001).

The livers of the *Lama4^−/−^* mice on standard diet did not display signs of liver steatosis after 40 weeks (File S1) suggesting that the decreased body fat in *Lama4^−/−^* mice does not represent a form of lipodystrophy. On a high-fat diet *Lama4^−/−^* mice only had very mild steatosis (File S1).

### Food consumption

In order to evaluate whether the observed differences in weight and body composition resulted from lower food consumption, food intake was quantified weekly. There was no difference in the total amount of food consumed between *Lama4^−/−^* and wild-type control animals on either standard (P = 0.418) or high fat diet (p = 0.098) (Figure 2C, D). These results suggest that the weight differences observed did not result from hypophagia in the *Lama4^−/−^* mice.

### Adipose Tissue Structure

*Lama4^−/−^* and age matched wild-type control animals 13 to 15 week on a standard diet were used to further examine adipose tissue structure and function. This time range was selected because it is prior to any statistically significant weight differences between *Lama4^−/−^* and control animals, allowing for the examination of adipose tissue function without confounding results due to obesity. At the time of sacrifice there were no differences in total animal mass between *Lama4^−/−^* and control animals (*Lama4^−/−^* 25.93±0.77 g, n = 4; *Lama^+/+^* 26.60±2.35 g, n = 4; *p* = 0.80).

**Figure 3:**
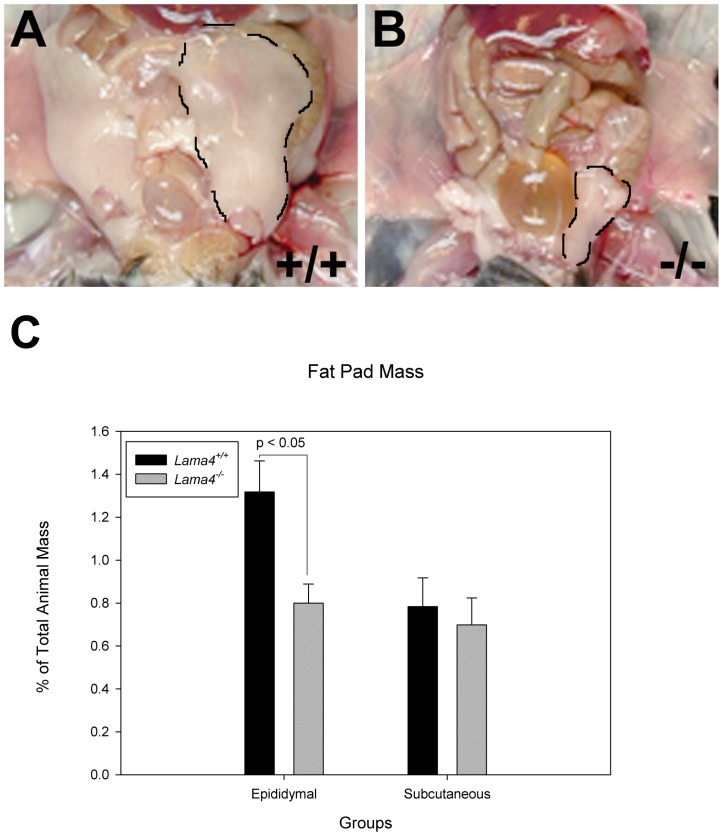
The *Lama4* ^−/−^ mice had reduced fat depots. The epididymal fat is delineated in an opened 40 week old male wild-type control mice (A) and a male *Lama4* ^−/−^ mice is shown in (B). Normalized fat pad depot mass as a % of total animal mass (C). Epididymal fat pad mass in *Lama4^−/−^* mice was significantly lower than *Lama4^+/+^* mice (p<0.05). Age matched animal *Lama4^−/−^* and *Lama4^+/+^*, n = 4 for epididymal and subcutaneous. Error bars represent standard error.

Epididymal and subcutaneous adipose tissue were harvested from the mice (Figure 3A, B). The normalized percentage mass of *Lama4^−/−^* mice's epididymal adipose tissue was significantly less than control mice (*Lama4^−/−^* 0.80±0.09%, n = 4; *Lama^+/+^* 1.32±0.14%, n = 4; p = 0.022) (Figure 3C). The mass of *Lama4^−/−^* mice's epididymal adipose tissue was less than control mice (*Lama4^−/−^* 0.21±0.03 g, n = 4; *Lama^+/+^* 0.36±0.07 g, n = 4; *p* = 0.10). Interestingly, no differences in mass were observed between subcutaneous adipose tissue mass (*Lama4^−/−^* 0.18±0.04 g, n = 4; *Lama^+/+^* 0.21±0.06 g, n = 4; *p* = 0.69). These results indicate that prior to any phenotypic observations in total animal weight gain, epididymal volume was reduced in *Lama4^−/−^* mice.

**Figure 4:**
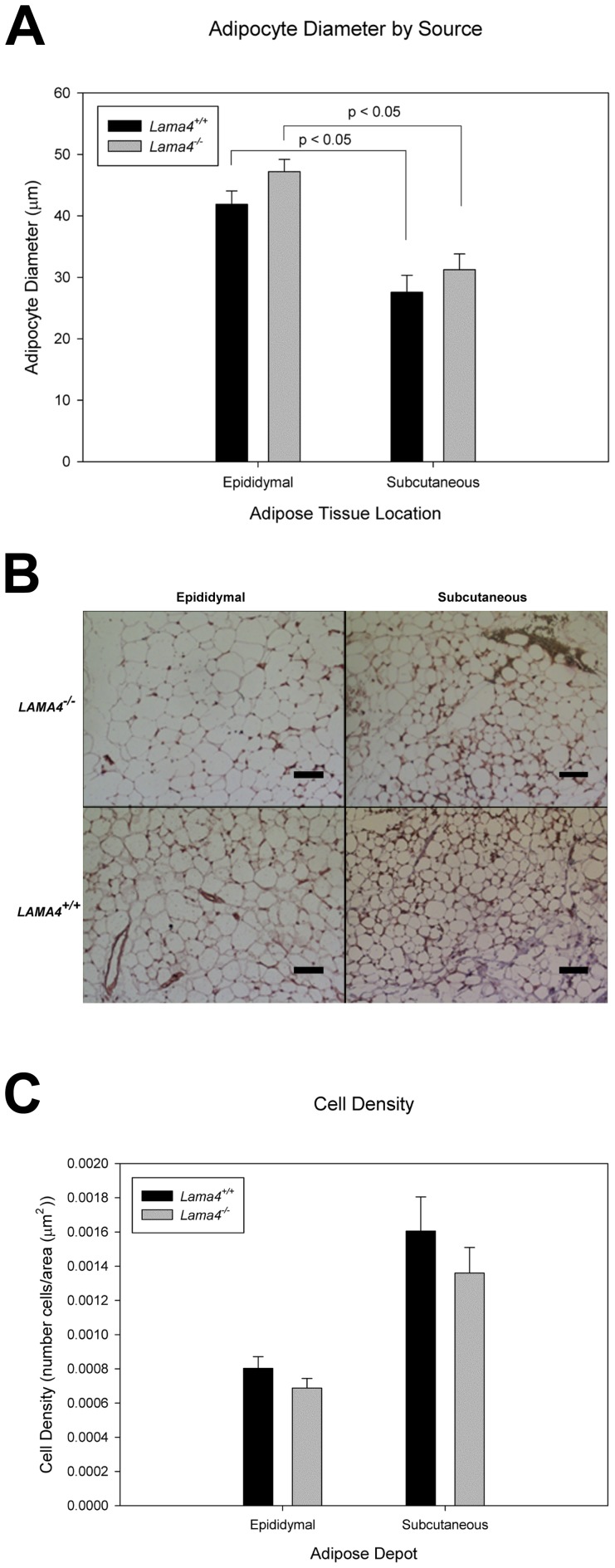
Adipocyte diameter in subcutaneous and epididymal adipose tissue depots for age matched *Lama4^−/−^* and *Lama4^+/+^*mice (A). For epididymal n = 4 and subcutaneous n = 5, standard error shown as the error bars. There is a statistical difference between epididymal and subcutaneous (P<0.05). Histology images of epididymal and subcutaneous *Lama4^−/−^* and *Lama4^+/+^*, scale bar equals 300 µm (B). Adipocyte cell density in subcutaneous and epididymal adipose tissue depots for age matched *Lama4^−/−^* and *Lama4^+/+^*mice (C). For epididymal n = 4 and subcutaneous n = 5, standard error shown as error bars.

Histomorphometrix analysis was used to further analyze adipose tissue in the different depots. Mean adipocyte size determined from histological stains was greater in epididymal adipose tissue than in the subcutaneous adipose tissue depots in both types of mice (Figure 4B). In both subcutaneous and epididymal depots the mean adipocyte size was greater in the *Lama4^−/−^* mice (Figure 4A). Cell density (number of cells per area) was lower in both epididymal and subcutaneous adipose tissue depots for *Lama4^−/−^* mice relative to wild-type controls (Figure 4C).

### Adipose Tissue Function

**Figure 5:**
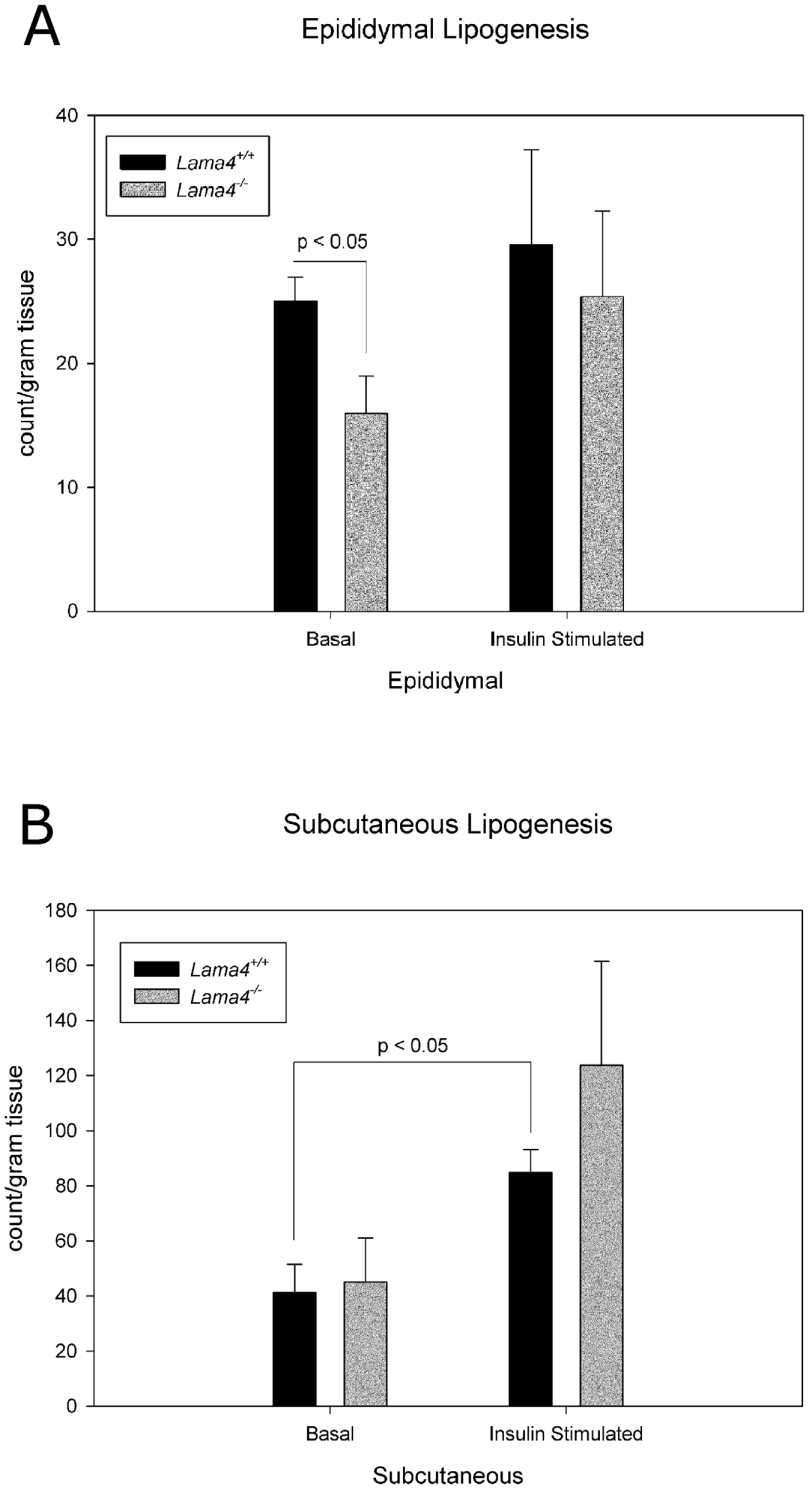
Lipogenesis levels in adipocytes from epididymal (A) and subcutaneous (B) adipose tissue in age matched *Lama4^−/−^* and *Lama4^+/+^*mice. Epididymal fat pad basal lipogenesis is statistically significant between *Lama4^−/−^* and *Lama4^+/+^*mice (p<0.05). Subcutaneous fat pad in *Lama4^+/+^*mice had statistically significant increase in lipogenesis between basal and insulin stimulated (p<0.05). The epididymal and subcutaneous n = 4. Error bars represent standard error.

The reduced adiposity may be an indication of altered metabolic function. Adipose tissue function was examined by quantifying basal and insulin-stimulated lipogenesis in adipose tissue isolated from *Lama4^−/−^* and wild-type control mice (Figure 5A, B). The epididymal depot of *Lama4^−/−^* mice exhibited impaired basal lipogenesis levels (Figure 5A). Interestingly, there were no differences in lipogenesis between subcutaneous adipocytes isolated from *Lama4^−/−^* and wild-type control mice (Figure 5B).

## Discussion

Differentiated adipocytes are surrounded by a thin BM layer. ECM in general, and BM specifically, have been shown to regulate cell behavior in a number of tissues and organs [3–7]. However, there is little knowledge of its role in adipocyte behavior. In this study, we used mice absent in a specific BM protein, laminin α4, to investigate its role in adipose tissue. Using these mice we found a profound influence of laminin α4 on the expansion and function of adipose tissue

*Lama4^−/−^* mice were found to gain weight at a much slower rate than wild-type control mice. This occurred during normal aging and was more pronounced on a high fat diet. In fact, *Lama4^−/−^* mice did not exhibit any differences in weight whether on normal chow or a high fat diet. Differences from wild type animals did not result from reduced food intake, and a reduction in adipose tissue was observed grossly in the mice deficient in laminin α4. While these results clearly showed that *Lama4^−/−^* mice have reduced adiposity, it was initially not clear if this resulted in normal or impaired adipose tissue function. Under both normal and high fat diets, livers in the *Lama4^−/−^* mice exhibited little steatosis, suggesting that the decreased body fat did not represent a form of lipodystrophy.

Laminin α4 is present around cells in the kidney, vasculature and muscle and has been shown to regulate different cell behaviors [17]–[26]. In order to further evaluate the role of laminin α4 specifically we isolated adipocytes from these tissues and analyzed their metabolic function. Surprisingly, we found that decreased laminin α4 leads to impaired adipocyte lipogenesis. Insulin stimulated lipogenesis resulted in increased lipogenesis in adipocytes from both *Lama4^−/−^* and wild-type control animals indicating that insulin responsiveness appears to be intact, but basal lipogenesis levels were lower in *Lama4^−/−^* mice. Interestingly, this difference was depot specific. Adipocytes isolated from epididymal adipose tissue exhibited impaired lipogenesis compared to wild-type controls while the subcutaneous adipose tissue depot had similar lipogenesis to wild-type control. This depot specific impairment is interesting and is further supported by the fact that epididymal adipose tissue depots in *Lama4^−/−^* mice significantly are smaller in volume than wild-type control animals at 14 weeks. This difference was observed prior to any measurable differences in total body weight. While the total amount of epididymal adipose tissue in *Lama4^−/−^* mice was lower than wild-type controls, the individual adipocytes were larger in *Lama4^−/−^* mice relative to wild-type controls. The subcutaneous depots were similar in both total volume and cell size in comparisons between *Lama4^−/−^* and wild-type control mice. Overall, these results suggest an impaired function in adipose tissue from mice absent in laminin α4 and that this impairment is manifested primarily in the epididymal depot.

This study shows a profound role for laminin α4 on adipose tissue expansion and function. However, the mechanism underlying this influence is unclear. There is very little knowledge of the role of the ECM on adipocyte behavior. Cell culture and tissue engineering studies have shown that ECM substrata influence adipocyte behavior. In 2D culture “laminin” was found to be more potent than type IV collagen, fibronectin or type I collagen at promoting differentiation of preadipocytes [29]. This study did not distinguish which the specific laminin isoform used, but it is likely that this commercial laminin was purified from a mouse soft tissue tumor that has been shown to contain laminin 111 (α1:β1:γ1). Studies with laminin α4 are less common due to it only recently becoming available commercially. Preadipocytes have been shown to produce LN-411 (α4:β1:γ1) during induction to an adipocyte phenotype [14] and laminin α4-rich ECM mixtures isolated promote greater adipogenesis than ECM lacking α4 [30]–[32]. The α4 chain laminin could directly regulate adipocyte function through modulation of cell receptor signaling.

Laminins containing the α4 chain could also influence adipocyte behavior based on indirect effects on the local cell microenvironment. The BM structure appeared thicker in immunohistochemical stains and previous studies have shown that the vascular BM in *Lama4^−/−^* mice is altered structurally [15]. This structural change could reflect altered mechanical properties that may influence adipocyte function [33], [34]. In addition, LN-411, which contains the α4 chain, has a chondroitin sulfate chain [35], [36] which may function to sequester growth factors in the vicinity of a cell. This contributes to the regulation of growth factor availability and signaling near cell surface receptors [37]. Alterations in the laminin composition could alter the ability of the BM to bind growth factors and wild-type control the local concentration of regulatory factors. Laminin α5 has had the gene variants shown to contribute to human body composition and weight [27]. A small subset of extremely obese humans were found to have variants in the laminin genes [28]. Future studies are needed that can address the mechanism including alteration in energy expenditure for the lean phenotype observed in *Lama4^−/−^* mice. Additionally, genetic studies investigating laminin α4 in humans could lead to improved understanding of obesity.

## Conclusion

In summary, *Lama4^−/−^* mice exhibited reduced weight gain in response to both age and high fat diet. The mass and function of the adipose tissue in *Lama4^−/−^* mice were altered in a depot-specific manner. In particular, epididymal adipose tissue exhibited decreased mice and altered lipogenesis in *Lama4^−/−^* mice, but no differences were observed in the subcutaneous depot. The results suggest that: (1) impaired lipogenesis leads to diminished fat mass in *Lama4^−/−^* mice; (2) alterations in lipogenesis are adipose tissue depot-specific; and (3) specific ECM components dramatically influence adipose tissue function.

## Supporting Information

File S1 **Histology of livers of 10 months old male animals.** In *Lama4^−/−^* animals the histological picture was without signs of steatosis on standard diet, and on high-fat diet it was very mild.
(TIF)
