# Supplementary figures and images for: Laminin α4 Deficient Mice Exhibit Decreased Capacity for Adipose Tissue Expansion and Weight Gain
Source: PLoS One. 2014 Oct 13;9(10):e109854. doi: 10.1371/journal.pone.0109854 (PMC4195691; doi:10.1371/journal.pone.0109854)

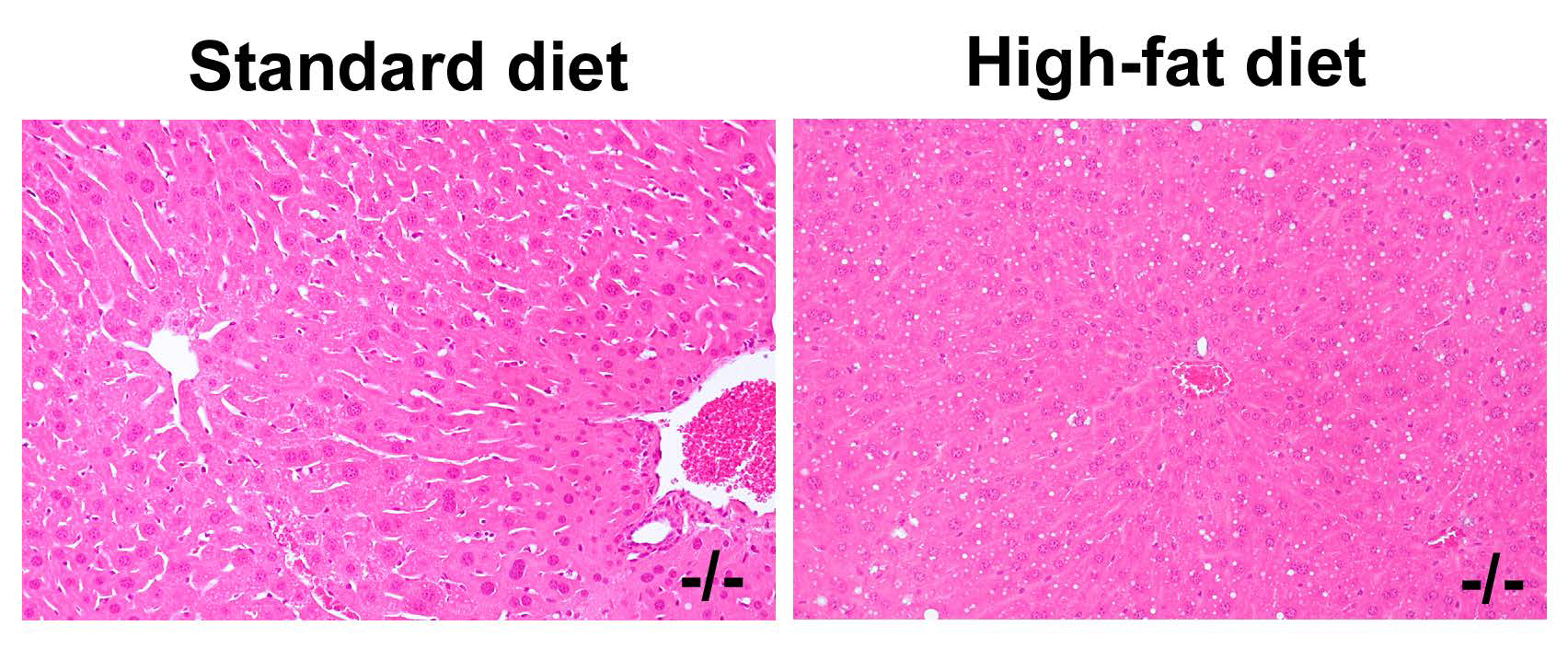

Supplement: File S1 — Histology of livers of 10 months old male animals. In Lama4−/− animals the histological picture was without signs of steatosis on standard diet, and on high-fat diet it was very mild. (TIF) [file pone.0109854.s001.tif]
